# Triactome: Neuro–Immune–Adipose Interactions. Implication in Vascular Biology

**DOI:** 10.3389/fimmu.2014.00130

**Published:** 2014-04-08

**Authors:** George Nikov Chaldakov, Marco Fiore, Peter I. Ghenev, Jerzy Beltowski, Gorana Ranćić, Neşe Tunçel, Luigi Aloe

**Affiliations:** ^1^Laboratory of Cell Biology, Department of Anatomy and Histology, Medical University, Varna, Bulgaria; ^2^Institute of Cellular Biology and Neurobiology, National Research Council, Rome, Italy; ^3^Department of General and Clinical Pathology, Medical University, Varna, Bulgaria; ^4^Department of Pathophysiology, Medical University, Lublin, Poland; ^5^Department of Histology and Embryology, University Medical Faculty, Niš, Serbia; ^6^Department of Physiology, Medical Faculty, Eskişehir University, Eskişehir, Turkey

**Keywords:** adipose tissue, adipokines, atherosclerosis, lymphocytes, mast cells, NGF, BDNF, perivascular nerves

## Abstract

Understanding how the precise interactions of nerves, immune cells, and adipose tissue account for cardiovascular and metabolic biology is a central aim of biomedical research at present. A long standing paradigm holds that the vascular wall is composed of three concentric tissue coats (*tunicae*): intima, media, and adventitia. However, large- and medium-sized arteries, where usually atherosclerotic lesions develop, are consistently surrounded by periadventitial adipose tissue (PAAT), we recently designated *tunica adiposa* (in brief, adiposa like intima, media, and adventitia). Today, atherosclerosis is considered an immune-mediated inflammatory disease featured by endothelial dysfunction/intimal thickening, medial atrophy, and adventitial lesions associated with adipose dysfunction, whereas hypertension is characterized by hyperinnervation-associated medial thickening due to smooth muscle cell hypertrophy/hyperplasia. PAAT expansion is associated with increased infiltration of immune cells, both adipocytes and immunocytes secreting pro-inflammatory and anti-inflammatory (metabotrophic) signaling proteins collectively dubbed adipokines. However, the role of vascular nerves and their interactions with immune cells and paracrine adipose tissue is not yet evaluated in such an integrated way. The present review attempts to briefly highlight the findings in basic and translational sciences in this area focusing on neuro–immune–adipose interactions, herein referred to as *triactome*. Triactome-targeted pharmacology may provide a novel therapeutic approach in cardiovascular disease.

*Today I want to tell you three stories from my life. That’s it. No big deal. Just three stories. The first story is about connecting the dots*.

***Steve Jobs***, from his Commencement address delivered on June 12, 2005 at the Stanford University, Stanford, CA, USA.

## Prolog

At the beginning of this century the Human Genome Project was finalized estimating over 30,000 genes encoding more than 100,000 functionally distinct proteins. As happened usually, one solved problem delivered many unsolved ones. Thus in the postgenome time, many “-ome” projects have emerged including proteome, transcriptome, interactome, metabolome, adipokinome, secretome, exposome, connectome so much numerous to be listed. Perhaps, this prompted Jeff Lichtman and Joshua Sanes to entitle one of their connectome articles *Ome sweet ome* [*Curr Opin Neurobiol* (2008) **18**:346–53].

The present review *is about connecting the dots* from neuroimmunology, adipobiology, and vascular biology into the hypothesis of neuro–immune–adipose interactions, herein designated *triactome*.

## Vascular Wall

Traditional view considers that the arterial wall is composed of three concentric tissue coats (*tunicae*): intima, media, and adventitia. However, in 1991, Soltis and Cassis studying rat aorta contractility wrote: “Virtually every blood vessel in the (human) body is surrounded to some degree by adipose tissue” (1). Forgotten, periadventitial adipose tissue (PAAT) has emerged again in the beginning of the 2000s (2–6). Indeed, large- and medium-sized arteries, where usually atherosclerotic lesions develop, are consistently surrounded by PAAT, recently conceptualized as fourth, outermost vascular coat, that is, *tunica adiposa* (hereafter also termed adiposa, like intima, media, and adventitia) (7, 8). Like epicardial adipose tissue (EAT) (9) and that around other internal organs (10), adiposa is not separated by a fascia from the underlying tissue, thus a bidirectional pathway between adiposa and other vascular coats is available for diffusible signals.

**Figure 1:**
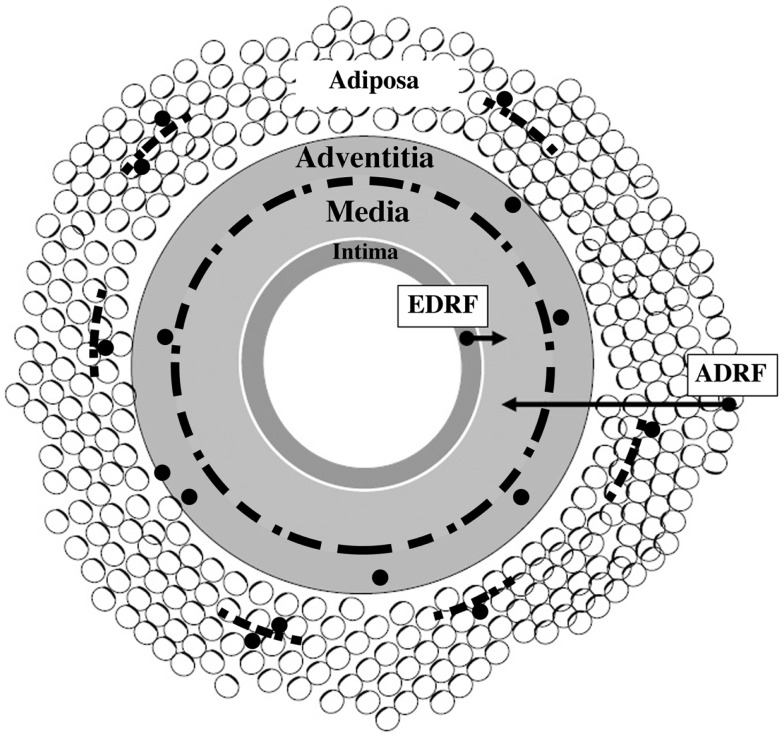
**Schematic presentation of vascular wall composed of four tissue coats (*tunicae*): intima, media, adventitia, and adiposa**. Arrows show that *tunica media* is a target for at least two vasorelaxing factors, endothelium-derived relaxing factor (EDRF) and adipocyte-derived relaxing factor (ADRF), respectively. Discontinuous black line positioned at the adventitia–media border illustrates perivascular nerves. Small-sized discontinuous black lines located in *tunica adiposa* indicate adipose nerves. Black granules (except those linked to arrows) illustrate immune cells – their association with nerves and adipocytes is also depicted. Modified from Ref. (14).

As depicted in Figure 1, the perivascular nerves are positioned at the media–adventitia border. Vascular as well as adipose tissue are extensively innervated by sympathetic nerves (11, 12), and their density correlates with the presence of nerve growth factor (NGF) (see below). The artery wall has the capacity to undergo remodeling in response to long-term changes or injuries. This is a process of structural rearrangement that involves cell growth, death, migration, phenotypic modulation, and secretion of extracellular matrix molecules by secretory phenotype smooth muscle cells (SMCs) (13).

## Vascular Disease

Cardiovascular disease (CVD) is the number one cause of death globally, and the major CVD’s phenotypes are atherosclerosis and hypertension. The raised (occlusive) intimal lesions are classically referred to as atherosclerotic plaques.

The inflammatory nature of atherosclerotic plaques was first described by Rudolf Virchow in 1858. Onward, since 1933 Nikolai Anitchkov’s lipid deposition and the 1970s Russell Ross’ proliferation of SMC in response to endothelial injury (15) were dominant concepts explaining the origin and development of atherosclerosis. However, in the mid 1980s, Göran Hansson has discovered immune cells (T cells and macrophages) in the human atherosclerotic plaque, hence created a *paradigm shift* in atherosclerosis research – the process of atherogenesis is governed by immune-mediated inflammatory mechanisms (16).

In brief, atherosclerosis is a progressive chronic lipid- and insulin resistance-driven inflammatory disease (15–19). This view however is mainly intima-centered, hence traditional pathological observations have given insight preferentially to the development of intimal lesions/luminal loss and respectively atherosclerotic plaque vulnerability, leading to myocardial infarction, stroke, and/or lower limb ischemia. In effect, the intima has been – for more than a century – considered the most important vascular area involved in atherogenesis. As a sequela, the intima–media thickness became an accepted measure of structural arterial remodeling and a strong predictor of atherosclerosis. However, it is unlikely that such one-direction road may solely travel the whole multiplex network like that of atherogenesis. Arguably, an interactive hypothesis was proposed, which appreciated the significance of all structural components of the artery wall including PAAT (2–4, 7).

Nowadays, paradigms defining the cell biology of vascular diseases are the following: (i) the hypertensive vascular wall is characterized by hyperinnervation-associated medial thickening due to SMC hypertrophy/hyperplasia, whereas (ii) the atherosclerotic vascular wall is characterized by intimal thickening, medial atrophy, and adventitial and adipose remodeling including the reduced expression of perivascular nerves (20, 21). Of note, a significant increase in the presence of links between perivascular nerves and adventitial mast cells was demonstrated in atherosclerotic coronary vessels (22, 23); whether this may be the case with adipose nerves and mast cells remain to be examined.

This brief description will be followed by a story about four *dots* and finally by their connection resulting in triactome hypothesis in vascular biology.

## Dot 1

### Neuroimmunology: Neurotrophins are not solely for neurons

Life at the cellular level requires growth-promoting trophic support and immune sensing. One of the biggest recent achievements of neurobiology is the study on neurotrophic factors. The neurotrophins are exciting examples of these factors.

At the end of the nineteenth century it was envisaged by Santiago Ramon y Cajal but has not been proved that the nerves require trophic support. By a rare combination of scientific reasoning and intuition, the proof was obtained by Rita Levi-Montalcini, Viktor Hamburger, and Stanley Cohen in the early 1950s in Saint Louis, MO, USA, where the first cell growth factor, namely NGF, was discovered (24). This was embodied in a conceptual framework well known now as neurotrophic (nerve–effector interaction) theory. It reveals a pivotal role of effector (target) cells in the control of neuronal differentiation, survival and function via production of NGF, and other neurotrophic factors. Around 15 years ago, one more component, the immune cell, was incorporated into the heart of nerve–effector interactions (biactome) to become neural–immune–effector (NIE) interactions (25, 26). The present triactome may thus be considered a variation on general theme of NIE.

The neurotrophin family of proteins consisted of NGF, brain-derived neurotrophic factor (BDNF), neurotrophin-3 (NT-3), NT-4/5, NT-6, and NT-7. Neurotrophins mediate their effects via ligation of: (i) pan-neurotrophin receptor, p75^NTR^, and (ii) receptor tyrosine kinase (tropomyosin-related kinase) (Trk), namely, TrkA (for NGF), Trk B (for BDNF and NT-4), and TrkC (for NT-3) (27, 28).

As often occurs, the framework of an initial concept of the physiological role of a newly discovered molecules extends in the light of emerging findings. This was also the case with NGF. During some 30 years after its discovery, there have been few reasons given to indicate that NGF acts on non-neuronal cells. Thus, it was remarkable to discover that treatment of newborn rats with NGF caused a systemic increase in the number of mast cells (29). This seminal finding paved the road of new research field, neuroimmunology (30, 31). Moreover, NGF and BDNF are synthesized, stored, and released not only by directly innervated cells but also by immune cells (32, 33) as well as other cell types (Table 1).

**Table 1:** **Cellular targets and sources for neurotrophins as potentially related to atherogenesis^a^**.

Immune cells	Other cells
Mast cells	Endothelial cells
Lymphocytes	Vascular smooth muscle cells
Macrophages	Fibroblasts/myofibroblasts
Dendritic cells	Platelets
Neutrophils	Adipocytes
	Perivascular nerves

*^a^From Ref. (34)*.

Taken together, NGF and its relative molecules are mediators of multiple biological phenomena in health and disease, ranging from the neurotrophic through immunotrophic and epitheliotrophic to metabotrophic effects. The evidence indicates that not only at neuroimmune, but also at cardiometabolic level life requires metabotrophic factors (those improving glucose, lipid, and adipokine metabolism) such as NGF and BDNF (35, 36).

Furthermore, hypertension has recently been recognized as an immune disorder and accumulating evidence suggests that interactions between the sympathetic nerves, renin–angiotensin system, and immune cells play a role in blood pressure regulation (37, 38).

## Dot 2

### Adipoimmunology: Adipose tissue-associated immune cells

#### Lymphocytes, macrophages, and mast cells

The evaluation of interactions of adipose tissue with a variety of immune cells is becoming one of the challenging topics of current biomedical research. This may elevate our knowledge about various physiological and pathological processes such as inflammation and metabolism and related disorders (34, 39–47).

White blood cells are able to home in extralymphoid peripheral tissues, including adipose tissue – here, chemokines and their receptors are critical factors in such a trafficking process, the accumulation of lymphocytes and macrophages around dying adipocytes forming “crown-like structure,” a histological signature of white adipose tissue (WAT) in obesity (45, 46).

Mast cells were first described in 1878 by Paul Ehrlich (1854–1915) in his doctoral thesis “Contribution to the Theory and Practice of Histological Staining” [see Ref. (48)]. Ehrlich named these cells *Mastzellen*, meaning “well-fed cells,” because they had high numbers of cytoplasmic granules. He observed that mast cells were commonly located in connective tissue near blood vessels and nerves, as well as in inflammatory and tumor lesions. Mast cells are phenotypically and functionally versatile effector cells that have been traditionally associated with the immunoglobulin E-mediated allergic response. However, recent studies implicate these cells in the regulation of multiple processes such as inflammation, fibrosis, angiogenesis, fibrinolysis, hemostasis, and neuroimmune interactions, which could be associated with various immune inflammatory diseases (40, 49–51), hence being metaphorically dubbed *master cells* by Steve Galli (52). Supportively, Cromolyn and Ketotifen, two common mast cell stabilizers used in human allergic diseases, reversed pre-established obesity and diabetes in mice (53). In *ob/ob* mice (leptin deficiency-induced obesity) compared to lean controls, adipose mast cells are distributed differentially (54). Excitingly, a genetic connection between mast cells and blood lipids was recently established (17). Data of vascular and adipose mast cells are further discussed below.

Note that pioneering findings for the importance of paracrine interactions between adipose tissue and immune cells were provided by Caroline Pond and her colleagues in the 1990s (55), thus opening a new research field, adipoimmunology.

## Dot 3

### Vascular biology: Do not ignore perivascular nerves and adventitial immune cells

In 1962, Schwartz (56) wrote with respect to the presence of adventitial mononuclear cell infiltration: “It is perhaps surprising that such prominent cellular accumulation should have received so little attention. Nevertheless, since cellular infiltration of the adventitia shows such a constant relationship to the presence and degree of plaque formation, it should not be disregarded.” This and other related works (56) have been largely ignored, and the atherosclerosis research for a long time has been, as mentioned above, focused on the intimal lesion (just one more example of *epistemological paralysis*). However, the observation that adventitial injury (including that of perivascular nerves) alone can lead to intimal thickening is an evidence for the dynamic interaction between adventitia and intima (20, 21); this is also the case with adiposa–media–intima interactions (see below).

### Coronary restenosis

In 1983, at the seminar organized by Dr. George Pappas (Department of Anatomy, Medical School, University of Illinois, Chicago, IL, USA), one of us (George Nikov Chaldakov) delivered a lecture entitled “The fine structure of secretory-state SMC and their possible role in occlusive arterial diseases.” During the discussion, the question whether some adventitial fibroblasts may migrate to the intima was raised. The answer of the author was “I do not know. It seems impossible.” However, what seemed “impossible” in 1983 was proven possible in 1996 when Shi et al. [Ref. (57), also see Ref. (58)] and Wilcox and Scott (56) summarized their results indicating that the adventitial fibroblasts proliferate and modulate their phenotype to myofibroblasts migrating to the intima of balloon-injured coronary arteries, thus contributing to the neointimal formation. Further, it was recently suggested that neoadventitial formation, consisted of fibrotic tissue and immune cells, could play an important role in coronary restenosis by circumferential scar-like contraction, which may cause luminal narrowing (59). These data suggest an important role of adventitial fibroblasts and immune cells, and bring into question the sole contribution of SMC to neointimal thickening in coronary restenosis.

### NGF, p75^NTR^ and mast cells in human coronary atherosclerosis

**Figure 2:**
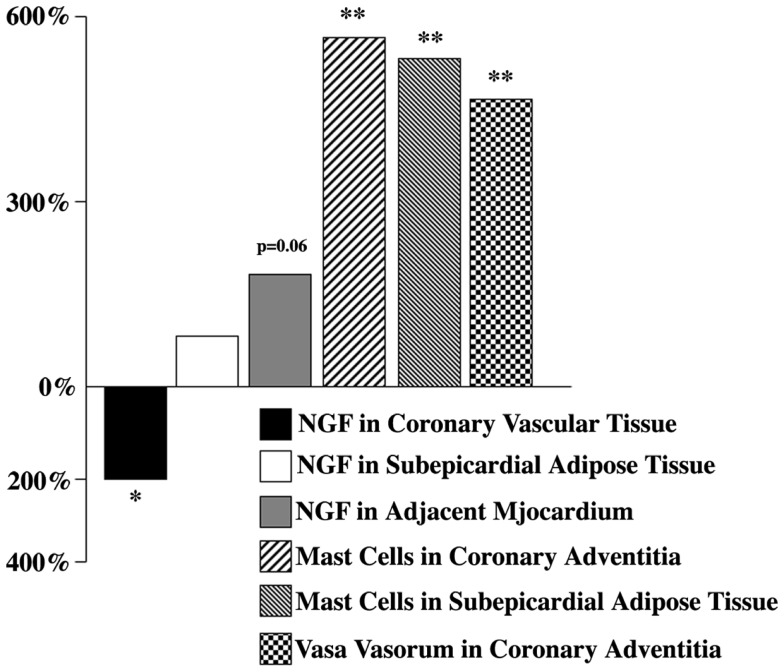
**Nerve growth factor, mast cell, and vasa vasorum changes in selected human atherosclerotic cardiac tissues expressed as percentage of controls**. Modified from Ref. (34).

In 2001, the first results about altered amount of NGF in the human coronary vascular wall affected by advanced atherosclerosis have been published [Ref. (35, 60), also see Ref. (14, 30, 34, 61)]. The expression of NGF and its receptor p75^NTR^ in the surrounding subEAT (coronary *tunica adiposa*) has been examined simultaneously [Ref. (34, 60), also see Ref. (62)]. It was found that the reduced NGF level was accompanied by an elevated amount NGF both in subEAT and the adjacent myocardium (Figure 2). Immunohistochemical analyses of coronaries revealed that coronary vascular wall, particularly the adventitia and subEAT, expressed a stronger p75^NTR^ immunoreactivity in atherosclerotic compared to control arteries (60).

Since mast cells are known to be a cellular component of the coronary artery and, as indicated above, these cells not only respond to NGF action (29), but also produce and release NGF (32), the presence and distribution of mast cells in atherosclerotic and control coronaries have been also examined. In atherosclerotic vessels, mast cells (number/millimeter square) were significantly increased both in adventitia and subEAT (Figure 2). Whether these mast cell populations, via their potential to synthesize and release NGF, attempt to compensate the reduced NGF in the coronary wall, remains to further be studied.

Although “many roads lead to atheroma,” the prevailing hypothesis at present is the Russell Ross’ response-to-injury hypothesis (15), which states that atherosclerosis is an inflammatory disease that involves several aspects of wound healing. Importantly, at cell biological level wound healing may be considered one of the most remarkable conceptual contributions of Russell Ross (his pre-atherogenesis studies were namely on skin biology). Therefore one may envisage atherosclerotic intimal lesions as vascular wound. Of note, one of us (Luigi Aloe) provided clinical results of therapeutic contribution of NGF in skin and corneal wound healing (63) and thus raises a pressing question of whether this may also be the case with vascular wound, that is, the atherosclerotic plaque (15).

## Dot 4

### Adipobiology: WAT is secretory whereas BAT thermogenic organ

**Figure 3:**
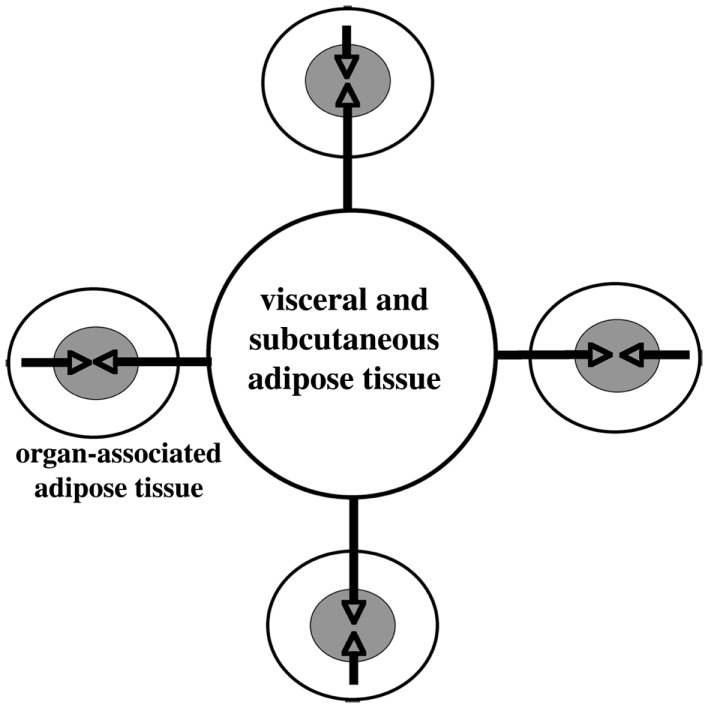
**Schematic illustration of large adipose depot (visceral and subcutaneous adipose tissue) and small adipose depots (organ- associated adipose tissue)**. Dual action of adipokines, via endocrine pathway (long arrows) and via paracrine pathway (short arrows) on the adipose tissue-associated organs, is depicted. Modified from Ref. (66).

Accumulation of adipose tissue in the visceral and subcutaneous abdominal tissue, also around internal organs (Figure 3), is a major risk factor for the development of numerous disorders including cardiovascular and metabolic diseases. Recently, *metaflammation* (metabolically induced inflammation) has emerged as a pivotal process involved in the clustering of those disorders (64, 65).

Adipose tissue is very plastic tissue, being constantly remodeled along with weight gain and weight loss. It is a dynamic multicellular and matrix assemble composed of adipocytes, fibroblasts, immune cells, blood vessels, stem cells, and sympathetic nerve fibers (11, 12). There are two major subtypes of adipose tissue, WAT and brown adipose tissue (BAT).

By sending and receiving different types of protein and non-protein signals, adipose tissue communicates with many organs in the body, including the brain, thus contributing to the control of energy, lipid, and glucose homeostasis as well as inflammation, immunity, learning, and memory among many other biological functions (10). Fat mapping (adipotopography) is an emerging biomedical field dealing with localization and amount of adipose tissue in the human body – fat on the outside and fat on the inside. Jeffrey Bell and colleagues (67) have scanned nearly 800 people with magnetic resonance imaging (MRI) technique, aimed at obtaining map of WAT. The authors demonstrated that as many as 45% of women and nearly 60% of men scanned have normal scores of the body mass index (BMI, 20–25 kg/m^2^). These people are thin outside (TO), while actually have excessive levels of internal adipose tissue – they are fat inside (FI), hence TOFI phenotype of body fatness. Noteworthy, TOFI phenotype was also found among people who are professional models. TOFI may thus be considered a specific, “invisible” expression of *Homo obesus* (68) and recently introduced *Homo diabesus* (69).

In human body, while WAT stores energy, BAT has the ability to dissipate energy by producing heat. BAT-mediated increase in energy expenditure is realized by uncoupling respiration from ATP synthesis, via uncoupling protein 1 (UCP1), which is expressed in brown adipocytes, thus generating heat, a process known as adaptive thermogenesis (70, 71). Animal studies have shown that activation of BAT counteracts diet-induced weight gain and related disorders such as type 2 diabetes mellitus and metabolic syndrome (70, 71). Recently, the knowledge about WAT and BAT was enriched with their relatives, namely *brite* (brown in white) and *bruscle* (brown in skeletal muscle) adipocytes (72). Hence, brown adipobiology is emerging as a new challenge in biomedicine.

### Adipoparacrinology

In 1933, Smith and Willius (73) found that “in most instances, a definite relationship between the excess of epicardial fat and the degree of general obesity occurred,” suggesting a functional relationship between EAT and atherosclerosis of left anterior descending (LAD) coronary artery (73).

One of the biggest recent achievements in studying cardiometabolic diseases is associated with the “rediscovery” of an ignored tissue, the adipose tissue. Adipose tissue considered as a passive storage-releaser of lipids and heat by most cell biologists and pathologists for a long period of time, can no longer be neglected in almost any biomedical field. The last 19–20 years, that is, the time after Jeffrey Friedman’s discovery of leptin, have seen it rise above the horizon to become the root cause of a plethora of syndromes and diseases (66, 74–76). Such an adipocentric approach has revealed that while BAT is major thermogenic organ, WAT is the body’s largest endocrine and paracrine organ producing a dazzling number of adipokines, with NGF and BDNF being also produced from adipose tissue (62, 77–79).

Obese phenotype of WAT is featured by adipocyte hypertrophy and/or hyperplasia leading to hypoxia and invasion of immune cells, resulting in an increased production of pro-inflammatory adipokines (80). By contrast, the secretion of adiponectin, an adipokine with anti-inflammatory, anti-obesity, insulin sensitizing, and vasorelaxing, that is, metabotrophic activity (68, 69), is decreased in obesity and related vascular diseases (81–86).

One aspect of the role of *tunica adiposa*, also EAT, is whether they facilitate or inhibit the process of atherogenesis. It is know that the proximal segments of coronary arteries are surrounded by subEAT, and these are atherosclerosis-prone as compared to the distal, intramyocardial, adipose-free, and atherosclerosis-resistant coronaries (34, 73). However, when EAT is totally absent, as in congenital generalized lipodystrophy, coronary atherosclerosis can still occur, suggesting that a homeostatic presence of adipose tissue is required for coronary artery health, reminding the maxim “A little fat is good” or “Fatter is better?” (84). On the same vein: (i) the removal of PAAT enhances neointima formation after injury, which is attenuated by transplantation of subcutaneous adipose tissue (81), whereas (ii) the excision of LAD coronary EAT (adipectomy) decreases the progression of atherosclerosis, suggesting a positive correlation between coronary EAT and atherosclerosis (9). Obviously, there is very much more to learn about the biology of this fascinating tissue.

Whatever changes occur in *tunica adiposa*, little is known of whether they can be causally associated with atherogenesis or whether they are a paracrine reaction to the injury developing within other layers of the artery wall, particularly the adventitia. Given the key role of inflammation in the development of atherosclerotic lesions, what role might then adiposa play in the process of atherogenesis? As indicated above, the expansion of adipose tissue seen in obesity is associated with adipose inflammation leading to an imbalanced secretion including: (i) an enhanced release of pro-inflammatory adipokines, and (ii) a decreased release of anti-inflammatory (metabotrophic) adipokines (Table 2) as well as (iii) a disbalance in contractile and relaxing factors released from adiposa (Table 3). Such a yin-and-yang pattern of cell secretion requires a research aiming at: (i) the inhibition of secretion and/or receptor sensitivity of pro-inflammatory and vasocontractile adipose-derived mediators, and (ii) the stimulation of secretion and/or receptor sensitivity of anti-inflammatory (metabotrophic) and vasorelaxing adipose-derived mediators (87, 88).

**Table 2:** **Selected list of pro- and anti-inflammatory adipose-derived signals relevant to cardiovascular disease**.

Pro-inflammatory signals	Anti-inflammatory signals
Tumor necrosis factor-α	Adiponectin
Interleukin-1β, -18/inflammasome	Interleukin-10
Hypoxia-inducible factor 1α	Nerve growth factor
MIP-1 (CCL2)	Interleukin-1 receptor antagonist
Leptin	Brain-derived neurotrophic factor
RANTES (CCL5)	Humanin^a^
Fractalkine (CX3CL1)	Irisin
Interleukin-8 (CXCL8)	Apelin, Otopetrin 1
Resistin	Omentin, Chemerin
ROS	Resolvin D1
Acylation stimulating protein	
Netrin-1	
Profilin-1	

*^a^Humanin is not (yet) adipose-derived product; it is a peptide produced by mitochondria (89)*.

*MIP-1 (CCL2), monocyte chemoattractant protein (CCL2, cysteine–cysteine modified chemokine ligand 2); RANTES, regulated on activated normal T cell expressed and secreted; ROS, reactive oxygen species*.

*For references see the text, also Ref. (89–108). Connecting references (63, 92, 93), NGF might be implicated in the therapy of atherosclerotic lesion viewed as vascular wound (15)*.

**Table 3:** **Adipose tissue-derived mediators controlling vascular tone^a^**.

**Vasodilators**
Nitric oxide (NO), adipocyte-derived relaxing factor, hydrogen sulfide (H_2_S), adiponectin, cardiac natriuretic peptide, adrenomedullin, visfatin, omentin
**Vasoconstrictors**
Superoxide anion, angiotensin II, endothelin-1, tumor necrosis factor-α

*^a^All components of renin–angiotensin system are also expressed in periadventitial adipose tissue, suggesting their paracrine involvement in the pathogenesis of atherosclerosis and hypertension (37, 87). Whether adipose-derived contractile mediators may contribute to the so-called “adventitial shrinkage” due to myofibroblast contraction in postangioplasty coronary restenosis (59), remains to be studied. For other mediators see Ref. (109–118)*.

Arguably, adipoparacrine activity is increasingly implicated in the pathogenesis of CVD. “So what does it mean if” (119) adipoparacrinology (10) and adipoimmunology (74) are indeed a biological rationale in vascular biology? First, in basic research, we should no longer disregard adventitia and adiposa, but preserve them in place and subject to a thorough examination; in other words, we need to keep open minds on *all* vascular coats. Second, echocardiography, computer tomography, MRI, and other non-invasive imaging of heart- and artery-associated adipose tissue may identify high-risk population susceptible to CVD (120, 121). Third, “non-touch harvesting technique” is an example of appreciation of adipoparacrinology in coronary artery bypass surgery (109, 110). Fourth, *tunica adipose* like adventitia (59) may represent a new target for *in situ* therapeutic applications.

## Connecting the Dots: Vascular Triactome

A central aim of neuroadipoimmunology is to map molecular interactions, in order to learn how they account for cardiovascular and metabolic biology and how alterations in them lead to disorders, including atherosclerosis and hypertension. However, until now we have paid less attention to the possible interactive talk in the vascular wall. When connecting the dots described above we may better understand which are the mediators in the suggested interactions between perivascular nerves, adventitial/adipose immune cells, and paracrine adipose tissue, herein designated vascular triactome. As indicated in Tables 2 and 3, the adipose-derived signaling molecules might be among the major players in the triactome. In effect, we may integrate the traditional “inside-out” (intimal) to an “outside-in” (adventitial and adipose) pathway in the pathogenesis of CVD. The present hypothesis may thus provide new insights into the therapy of these diseases.

*That’s it. No big deal. Just a triactome* (Figure 1). The future challenge is therefore to cultivate integrative thinking about how we can make triactome work for the benefit of cardiometabolic health.

## Conflict of Interest Statement

The authors declare that the research was conducted in the absence of any commercial or financial relationships that could be construed as a potential conflict of interest.
